# A before and after study of the impact of academic detailing on the use of diagnostic imaging for shoulder complaints in general practice

**DOI:** 10.1186/1471-2296-8-12

**Published:** 2007-03-27

**Authors:** Norm A Broadhurst, Christopher A Barton, Debra Rowett, Lisa Yelland, David K Matin, Angela Gialamas, Justin J Beilby

**Affiliations:** 1 Department of Orthopaedics, Finders University, Bedford Park, South Australia, Australia; 2 Discipline of General Practice, School of Population Health and Clinical Practice, University of Adelaide. Adelaide, South Australia. Australia; 3 DATIS, Repatriation Hospital, Daw Park, South Australia. Australia

## Abstract

**Background:**

The aim of this study was to assess the impact that Academic Detailing (AD) had on General Practitioners' use of diagnostic imaging for shoulder complaints in general practice and their knowledge and confidence to manage shoulder pain.

**Methods:**

One-to-one Academic Detailing (AD) for management of shoulder pain was delivered to 87 General Practitioners (GPs) in metropolitan Adelaide, South Australia, together with locally developed clinical guidelines and a video/DVD on how to examine the shoulder. Three months after the initial AD a further small group or an individual follow up session was offered. A 10-item questionnaire to assess knowledge about the shoulders was administered before, immediately after, and 3 months after AD, together with questions to assess confidence to manage shoulder complaints. The number of requests for plain film (X-ray) and ultrasound (US) imaging of the shoulder was obtained for the intervention group as well as a random comparison group of 90 GP's from the same two Divisions. The change in the rate of requests was assessed using a log Poisson GEE with adjustment for clustering at the practice level. A linear mixed effects model was used to analyse changes in knowledge.

**Results:**

In an average week 54% of GPs reported seeing fewer than 6 patients with shoulder problems. Mean (SD) GP knowledge score before, immediately after and 3-months after AD, was 6.2/10 (1.5); 8.6/10 (0.96) and; 7.2/10 (1.5) respectively (p < 0.0001). Three months after AD, GPs reported feeling able to take a more meaningful history, more confident managing shoulder pain, and felt their management of shoulder pain had improved. Requests for ultrasound imaging were approximately 43.8% higher in the period 2 years before detailing compared to six months after detailing (p < 0.0001), but an upward trend toward baseline was observed in the period 6 months to 1 year after AD. There was no statistically significant change in the rate of requests from before to after AD for plain-radiographs (p = 0.11). No significant changes in the rate of requests over time were observed in the control groups.

**Conclusion:**

These results provide evidence that AD together with education materials and guidelines can improve GPs' knowledge and confidence to manage shoulder problems and reduce the use of imaging, at least in the short term.

## Background

Shoulder pain is third following back and neck pain as a musculoskeletal reason for presenting to general practice in Australia. Approximately 10% of the adult population are expected to visit a General Practitioner (GP) for shoulder pain at least once in a lifetime [1,2].

Despite the finding that 50% of acute shoulder pain resolves in 8–10 weeks many patients present with the anticipation of having some kind of imaging [3,4]. There are a range of diagnostic imaging tests that can be used in the evaluation of shoulder pain including plain radiographs, arthrography, computed tomography, ultrasound, and magnetic resonance imaging [5]. The history and physical examination are keys to most shoulder pain diagnoses, particularly when used in combination. Often no imaging is required, or plain radiographs are the sole imaging study needed [5].

Over recent years the Diagnostic Imaging Section of the Australian Government Department of Health and Ageing identified a marked increase in the use of ultrasound imaging for problems relating to the shoulder. The increase in the number of ultrasounds performed for shoulder complaints have been at considerable cost to the Commonwealth Health Insurance Commission (HIC). In the year to April 2002, the cost increased by 110% to $12.9 million. The number of x-ray and ultrasound services performed in 2001–02 when compared with the previous year also increased by 110% to 152,073 investigations. GPs in Australia are unable to order magnetic resonance imaging (MRI) and so these costs are not considered here.

A project devised in three parts was initiated by the Musculoskeletal Ultrasound Project Team of the Australian Government Department of Health and Ageing and operated through the auspices of the Musculoskeletal Foundation of Australia. Stage I was to determine what was written by GPs on imaging request forms and then to compare this with the radiologist's report. This study found that about one third (34%) of requests for ultrasound imaging of the shoulder contained no tangible information to assist the radiological examination [6]. When a clinical diagnosis was provided by a GP, the degree of accuracy with the ultrasound findings was only 22%.

The results from Stage II [7] showed most imaging is ordered at the first visit of the patient presenting with shoulder pain, with the majority of patients having imaging ordered within five weeks of the first GP visit. Age (45–65 years), pain on activity, and shoulder pain present for ≥ 5 weeks increased the likelihood for a person to have imaging. Imaging (results similar in both Stage I and Stage II) revealed some pathology in 75% of patients.

The preliminary work completed in Stage I and Stage II provided good evidence that guidelines for imaging of shoulder complaints need to be established, and that GPs might benefit from education about management of shoulder problems. Stage III of the study presented here, sought to improve the assessment and management of shoulder complaints through the use of Academic Detailing to GPs, and to improve the knowledge and confidence of GPs to manage shoulder pain.

## Methods

Ethics approval was granted from the University of Adelaide Human Research Ethics Committee on 19 November 2003.

A 'before and after' study design was implemented involving two metropolitan divisions of General Practice (The Adelaide Central and Eastern (ACE) Division and the Western Division of General Practice (WDGP) who assisted the study team to recruit General Practitioners from the membership lists of these Divisions. GPs were eligible to participate if they were members in one of these Divisions and were working ≥ 0.5 full time equivalent (FTE). All GPs in the two Divisions who met the selection criteria were invited to participate.

GPs interested in participating in the study faxed a signed agreement to the Drug and Therapeutic Information Service (DATIS) at the Repatriation General Hospital, South Australia who made appointments with the GPs for Academic Detailing.

### Details of academic detailing

Two Specialists provided the Academic Detailing to GPs (NB and DM), at the GPs practice between December 2004 and March 2005.

Arrangements for Academic Detailing were coordinated through the DATIS Director (DR) and her staff, who are recognised experts in the field of Academic Detailing. The Detailers (NB and DM) provided one to one guidance to GPs on how to correctly assess the shoulder, and after having corrected the technique the GP was provided with education materials that included a DVD and guidelines (see Additional file 1). These were left with the GPs to be used during future patient assessments. The Detailing session lasted from 45 to 60 minutes.

At the initial AD visit each GP received:

• The reason for and the objectives of the study

• An evidence-based outline for shoulder imaging

• A video/DVD on the anatomy and examination of the shoulder. The Detailer used the video as a guide during an active demonstration on how to examine the shoulder.

• A reference information sheet to aid the management of patients with shoulder pain (see Additional file 1).

Three months after the initial AD a follow up session was offered either in small groups or on a one-to-one basis.

### Questionnaires

Demographic information about the GP and the practice they work in was collected by questionnaire. GPs then completed a ten-item questionnaire to determine knowledge about identifying and managing shoulder problems (see Additional file 2). The knowledge questionnaire was repeated immediately after the detailing, and again at the follow up visit when GPs also completed a 27-item questionnaire regarding confidence to manage shoulder problems and satisfaction with the AD model.

Questions asked referred to:

• The project generally;

• Assessment of study materials

• Assessment of the AD presentation

• Use of treatment modalities as a consequence of the Detailing.

### Imaging request data

The number of requests for plain film (Medicare Benefits Schedule Item number 57703) and US imaging (Medicare Benefits Schedule item number #55808) of the shoulder was provided by the HIC for all GPs on a month-by-month basis for the period January 2001 to March 2005.

Imaging requests were also obtained for a comparison group of 90 randomly selected GPs from the Adelaide Central and Eastern Division of General Practice (60 GPs), and the Western Division of General Practice (30 GPs) membership list.

### Statistical analysis

Data was initially entered into a Microsoft Access database and then transferred to SPSS version 12.0 for data cleaning purposes and preliminary data analysis. Frequency and range checks were carried out to ensure quality of data entry. Further statistical analysis was undertaken using SAS version 9.1 (Cary, NC, USA).

Demographic data and data collected from the confidence to manage shoulder problems questionnaire were analysed descriptively using frequencies. GP knowledge scores were analysed using a linear mixed effects model to investigate whether there was a change in knowledge across the three different testing times. Practice was included as a random effect and the model allowed for heterogeneity of variances across the testing times.

A log Poisson GEE was fitted to the HIC data of monthly imaging requests to investigate whether there was a change in the rate of requests made over four time periods of interest (period 1 = two year period before academic detailing, period 2 = month of Detailing, period 3 = six month period after Detailing, and period 4 = six month period after time period 3) and whether this change was different in the AD and control groups. Adjustment for clustering was made at the practice level. In order to define the four time periods of interest for a control GP, an 'Academic Detailing' month had to be assigned. This assignment was made randomly and ensured that within each division, an approximately equal percentage of GPs in the control and AD groups were assigned to each month in which AD was carried out. Results were adjusted for month since the data exhibited seasonal variation.

A p-value ≤ 0.05 was required for statistical significance.

## Results

Of the 369 ACE Division GPs 59 (16%) agreed to participate and out of  247 GPs in the WDGP 33 (13%) participated. Five GPs who initially responded to the invitation to participate either withdrew consent or were unable to complete the study leaving a total of 87 for the final analysis. Approximately half of GPs reported seeing fewer than 6 patients with shoulder problems a week. Further demographic details of the GPs can be found in Table 1.

**Table 1:** Demographic details of participating General Practitioners^1^.

		**ACE**^2^	**WDGP**^3^
		N (%)	N (%)

Gender	Male	34 (60.7%)	19 (61.3%)
	Female	22 (39.3%)	12 (38.7%)
GP Age	<35	2 (3.6%)	2 (6.5%)
	35–44	17 (30.4%)	5 (16.1%)
	45–54	25 (44.6%)	11 (35.5%)
	55–64	10 (17.9%)	10 (32.3%)
	65+	2 (3.6%)	0
Years in general practice	<= 5 years	3 (5.4%)	1 (3.2%)
	6–15 years	15 (26.8%	4 (12.9%)
	16–25 years	28 (50%)	17 (54.8%)
	26+ years	10 (17.9%)	7 (22.6%)
Number of full time equivalents at practice	Solo	7 (12.5%)	8 (25.8%)
	2 GPs	7 (12.5%)	8 (25.8%)
	3 GPs	9 (16.1%)	3 (9.7%)
	4 GPs	5 (8.9%)	4 (12.9%)
	5 or more GPs	27 (48.2%)	4 (12.9%)
	Other	1 (1.8%)	1 (3.2%)
Completion of Family Medicine	Yes	27 (48.2%	8 (25.8%)
Program or RACGP training program	No	29 (51.8%)	19 (61.3%)
	Current	0	2 (6.5%)
	Registrar		
Vocationally Registered	Yes	55 (98.2%)	29 (93.5%)
	No	1 (1.8%)	0
Number of patients with shoulder pain in an 'average' week	<6	31 (55.4%)	16 (51.6%)
	6–15	22 (39.9%)	11 (35.5%)
	16–25	3 (5.4%)	1 (3.2%)
	26+	0	1 (3.2%)
Approximate number of patients seen in a normal week	0–25	6 (10.7%	1 (3.2%)
	26–50	12 (21.4%)	4 (12.9%)
	51–75	15 (26.8%)	3 (9.7%)
	76–100	11 (19.6%)	8 (25.8%)
	101–125	9 (16.1%)	9 (29%)
	126–150	3 (5.4%)	4 (12.9%)

^1^Percentages do not always total 100 due to missing data

^2^ACE = Adelaide Central and Eastern Division of General Practice

^3^WDGP = Western Division of General Practice

**Figure 1:**
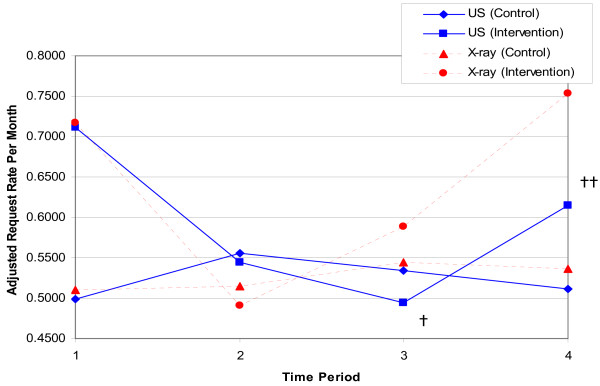
Adjusted rates of requests by time for plain shoulder x-ray (MBS Item 57703) and ultrasound (MBS Item 55808). Time Period 1 represents the two-year period before academic detailing; 2) represents the month of academic detailing; 3) represents the six-month period after academic detailing; 4) represents the six-month period after time 3. † – Time Period 3 compared with Time Period 1 in the Academic Detailing group (p < 0.01). †† – Time Period 4 compared to Time Period 3 in the Academic Detailing group (p = 0.036).

Figure 1 shows the time-adjusted rate of requests for plain x-ray and ultrasound made by GPs in the two years before the study (Time period 1), during the month of AD (Time period 2), in the six months immediately after AD (Time period 3), and in the next six-month period (Time period 4). There was no evidence to suggest a change in the rate of requests over the different time periods for plain x-ray in the intervention group compared to the control group (P = 0.11). Figure 1 also shows the time-adjusted rate of requests for ultrasound made by intervention and control group GPs. There is strong evidence to suggest that changes in the rate of requests over time periods were different for the AD and control groups (p = 0.02). The rate ratio describes the ratio of the rate of an event in one group to the rate in another group. In this context, we can use the rate ratio to compare the rate of requests between the AD and control groups, or during different time periods within the same group. Post hoc testing indicates that the estimated rate ratio comparing time 1 to time 3 for the AD group is 1.438 (p < 0.0001). This means that requests were approximately 43.8% more frequent during time 1 compared to time 3 in the academic detailing group, after adjusting for everything else in the model.

However, the estimated rate ratio comparing time 3 to time 4 is 0.80413 (p = 0.036). This means that requests were approximately 19.6% less frequent during time 3 compared to time 4 in the academic detailing group, after adjusting for everything else in the model. This indicates that in the period 6 months to 1 year after the academic detailing, the number of requests for ultrasound trended significantly upward toward baseline, but still tended to be less frequent than prior to the academic detailing, although this did not reach statistical significance (time 1 vs time 4, p = 0.13) (Table 2).

**Table 2:** Post Hoc testing of adjusted imaging rates for ultrasound (MBS item 55808).

Comparison	^1^Estimate	95% Lower Limit	95% Upper Limit	ChiSq	P-value	Sig
Period 1: control vs. academic detailing	0.701	0.470	1.045	3.04	0.081	NS
Period 2: control vs. academic detailing	1.022	0.597	1.749	0.01	0.938	NS
Period 3: control vs. academic detailing	1.080	0.690	1.689	0.11	0.737	NS
Period 4: control vs. academic detailing	0.831	0.565	1.223	0.88	0.348	NS
Control: period 1 vs. period 2	0.896	0.629	1.277	0.37	0.545	NS
Control: period 1 vs. period 3	0.933	0.758	1.150	0.42	0.517	NS
Control: period 1 vs. period 4	0.975	0.791	1.202	0.06	0.813	NS
Control: period 2 vs. period 3	1.041	0.714	1.519	0.04	0.834	NS
Control: period 2 vs. period 4	1.088	0.768	1.540	0.22	0.636	NS
Control: period 3 vs. period 4	1.045	0.846	1.289	0.17	0.685	NS
Academic detailing: period 1 vs. period 2	1.306	0.951	1.795	2.72	0.099	NS
Academic detailing: period 1 vs. period 3	1.438	1.254	1.649	26.94	<.0001	*
Academic detailing: period 1 vs. period 4	1.156	0.956	1.398	2.24	0.134	NS
Academic detailing: period 2 vs. period 3	1.101	0.807	1.501	0.37	0.544	NS
Academic detailing: period 2 vs. period 4	0.885	0.643	1.218	0.56	0.453	NS
Academic detailing: period 3 vs. period 4	0.804	0.656	0.986	4.39	0.036	*

Nb. No adjustment has been made for multiple comparisons

^1^The estimate represents the post hoc comparison Rate Ratio

NS – Not Significant

* Indicates p < 0.05.

There were no other statistically significant differences in the rate of requests, either comparing time periods within treatment groups or comparing AD and control at each time period (Table 2).

The mean (St Dev) GP knowledge before detailing was 6.2/10 (1.5). Immediately after the detailing, this rose to 8.6/10 (0.96). Three months after the detailing, knowledge remained higher compared to pre-test knowledge (7.2/10 (1.5). There was very strong evidence of a change in knowledge amongst the 87 GPs over the three testing times (p < 0.0001) and post hoc analyses revealed significant differences in mean scores between each pair of testing times (p < 0.0001 in each case). Knowledge was greatest immediately after academic detailing, and remained significantly higher than baseline 3 months after academic detailing.

There was no evidence to suggest that any of the demographic variables had an effect on the average test score, except for the total number of patients per week (p = 0.0496). However, the post hoc analysis did not reveal a clear relationship between the total number of patients per week and average knowledge score and this result is considered an artefact.

Three months after academic detailing most GPs reported feeling able to take a more meaningful history, felt more confident managing shoulder pain, and felt that their management of shoulder pain had improved (Table 3).

**Table 3:** GPs confidence to manage musculoskeletal problems three months after participating in academic detailing

Item		N (%)*
I found the visit helpful for increasing management skills	Strongly Agree	22 (26.5%)
	Agree	56 (67.5%)
	No Change	4 (4.8%)
	Disagree	1 (1.2%)
	Strongly Disagree	0 (0%)
I have been able to take a more meaningful history	Strongly Agree	7 (8.4%)
	Agree	62 (74.7%)
	No Change	14 (16.9%)
	Disagree	0 (0%)
	Strongly Disagree	0 (0%)
My examination process is better developed	Strongly Agree	9 (10.8%)
	Agree	65 (78.3%)
	No Change	9 (10.8%)
	Disagree	0 (0%)
	Strongly Disagree	0 (0%)
I am managing shoulder pain more confidently	Strongly Agree	6 (7.4%)
	Agree	60 (74.1%)
	No Change	15 (18.5%)
	Disagree	0 (0%)
	Strongly Disagree	0 (0%)
From the history and examination I can identify the area/structure of the pain more readily	Strongly Agree	6 (7.4%)
	Agree	57 (68.7%)
	No Change	20 (24.1%)
	Disagree	0 (0%)
	Strongly Disagree	0 (0%)
My management of shoulder pain has improved since the Detailing	Strongly Agree	10 (12%)
	Agree	62 (74.7%)
	No Change	11 (13.3%)
	Disagree	0 (0%)
	Strongly Disagree	0 (0%)

* % does not always total 100 due to missing data.

## Discussion

The major goal of this study was to reduce the number of referrals for shoulder imaging, and this was achieved in the first six months after academic detailing for ultrasound imaging of the shoulder, but not for x-ray imaging. However, over the next six month period this could not be sustained and the frequency of requests for ultrasound imaging returned toward pre-academic detailing levels. While these GPs had increased knowledge and were more confident in managing shoulder pain, this was in contrast to the trend toward baseline in the number of imaging requests seen 12 months after detailing.

An assessment as to why GP's gravitated to their pre-AD level of practice in respect to managing shoulder pain is needed. Failure to maintain the reduced use of ultrasound could be due to limited exposure to patients with shoulder pain; time restraints in general practice that might lead to regression after the initial encouragement; or the threat of patient dissatisfaction or even legal challenge, therefore unnecessary imaging is ordered in the absence of a confident assessment. The later point may reflect declining confidence in assessment with time, as indicated by the decay in knowledge, and increasing use of ultrasound six months to one year after the initial academic detailing.

While previous studies on musculoskeletal management have shown deficiencies in the management of patients with pain in general practice, this study has established potential cost savings in the short term, from reduced use of ultrasound imaging. A more detailed and concentrated educational program might be necessary to sustain changes observed in imaging use for management of shoulder problems.

Guidelines for managing some musculoskeletal problems are well known and have a proven cost benefit [8-12]. The cost savings per item number from this study based on a 40% decrease in 6 months translates to a saving of ~$7000 per doctor per year. The big question would be whether a detailed educational program on shoulder management would be cost effective and enduring?

### Limitations of the study

The primary limitation of this study is the low participation rate (16%  in ACE Division and 13% in the WDGP). Those GPs that did participate were self-selected and so it is possible that they had a special interest in musculoskeletal problems. An inspection of the number of imaging requests made per month indicate that GPs who volunteered for academic detailing requested more imaging than control group GPs who did not volunteer to participate in academic detailing. However, there was a large range in the number of patients that GPs reported as presenting with shoulder complaints in an average week. Hence, it is likely that some of the GPs in this study had a special interest in musculoskeletal problems, but clearly not all did. A second limitation is that we did not evaluate whether our intervention actually made the use of imaging more appropriate according to the guideline. A final limitation relates to repeated testing of knowledge. Since the same questions were asked at each testing time, there may have been a learning effect.

## Conclusion

Academic Detailing has proven to be a means of containing shoulder imaging use in the short term. Further research is required to determine if the increase in knowledge and confidence for managing shoulder problems translates into better clinical management as well as reducing cost in the long term, while not compromising health outcomes and quality of life.

## Competing interests

The author(s) declare that they have no competing interests.

## Authors' contributions

NB was involved in conception and design, gaining funding, he developed study materials including guidelines and Academic Detailing materials, performed Academic Detailing, oversaw day-to day management of the project, provided insight to data analysis, and helped draft the manuscript. CB provided day-to-day management of project, sought ethics approvals, recruited GP participants, entered and analysed data, provided insight to data analysis, and helped draft the manuscript. DR was involved in conception and design, provided advice on the Academic Detailing program, co-ordinated delivery of Detailing, and provided insight to data analysis. LY performed advanced statistical analyses and interpretation and helped draft the manuscript. DM helped develop Academic Detailing materials and performed Academic Detailing. AG was involved in conception and design, gaining ethics approval and recruiting GPs prior to going on maternity leave. JB was involved in conception and design, gaining funding and provided insight to data analysis. All authors read and approved the final manuscript.

## Pre-publication history

The pre-publication history for this paper can be accessed here:



## Supplementary Material

Additional File 1 A guideline for shoulder imaging developed in consensus by 6 orthopaedic surgeons. Guidelines developed for the study for use by GPs to manage shoulder pain. Click here for file

Additional File 2 Assessment of knowledge about musculoskeletal problems of the shoulder. The questionnaire used in the study to assess GP knowledge about management of shoulder pain. Click here for file

## References

[B1] Mitchell C, Adebajo A, Hay E, Carr A (2005). Shoulder pain: diagnosis and management in general practice.. BMJ.

[B2] van der Heijden GJ, van der Windt DA, Kleijnen J, Koes BW, Bouter LM (1996). Steroid injections for shoulder disorders: a systematic review of randomised controlled trials.. Br J Gen Pract.

[B3] van der Heijden GJ, Croft P, Brooks PM (1999). Shoulder disorders: a state of the art review. Bailliere’s Clinical Rheumatology.

[B4] van der Windt DA, Koes BW, Boeke AJ, Deville W, De Jong BA, Bouter LM (1996). Shoulder disorders in general practice: the prognostic indicators of outcome.. Br J Gen Pract.

[B5] Stevenson JH, Trojian T (2002). Evaluation of shoulder pain. The Journal of Family Practice.

[B6] Broadhurst N, Baghurst T, MacLaren S (2004). Ultrasound imaging for shoulder pain in general practice.. Aust Fam Physician.

[B7] Broadhurst NA, Gialamas A, McElroy HJ, Beilby JJ (2004). How do Australian GP’s manage shoulder dysfunction?. Aust Fam Physician.

[B8] Hoffman JR, Wolfson AB, Todd K, Mower WR (1998). Selective cervical spine radiography in blunt trauma: methodology of the national Emergency X-radiography Utilizatio Study (NEXUS). Ann Emerg Med.

[B9] Spitzer WO, Skovron ML, Salmi LR, Cassidy JD, Duranceau J, Suissa S, Zeiss E (1995). Scientific monograph of the Quebec Task Force on Whiplash-Associated Disorders: redefining "whiplash" and its management.. Spine.

[B10] Stiell IG, Greenberg GH, McKnight RD, Nair RC, McDowell I, Worthington JR (1992). A study to develop clinical decision rules for the use of radiography in acute ankle injuries. Ann Emerg Med.

[B11] Stiell IG, Greenberg GH, Wells GA, McKnight RD, Cwinn AA, Cacciotti T, McDowell I, Smith NA (1995). Derivation of  a decision rule for the use of radiography in acute knee injuries.. Ann Emerg Med.

[B12] Stiell IG, Wells GA, Vandemheen KL, Clement CM, Lesiuk H, De Maio VJ, Laupacis A, Schull M, McKnight RD, Verbeek R, Brison R, Cass D, Dreyer J, Eisenhauer MA, Greenberg GH, MacPhail I, Morrison L, Reardon M, Worthington J (2001). The Canadian C-Spine Rule for radiography in alert and stable trauma patients. JAMA.

