# Supplementary figures and images for: A before and after study of the impact of academic detailing on the use of diagnostic imaging for shoulder complaints in general practice
Source: BMC Fam Pract. 2007 Mar 27;8:12. doi: 10.1186/1471-2296-8-12 (PMC1851961; doi:10.1186/1471-2296-8-12)

**A guideline for shoulder imaging developed in consensus by 6 orthopaedic surgeons.**


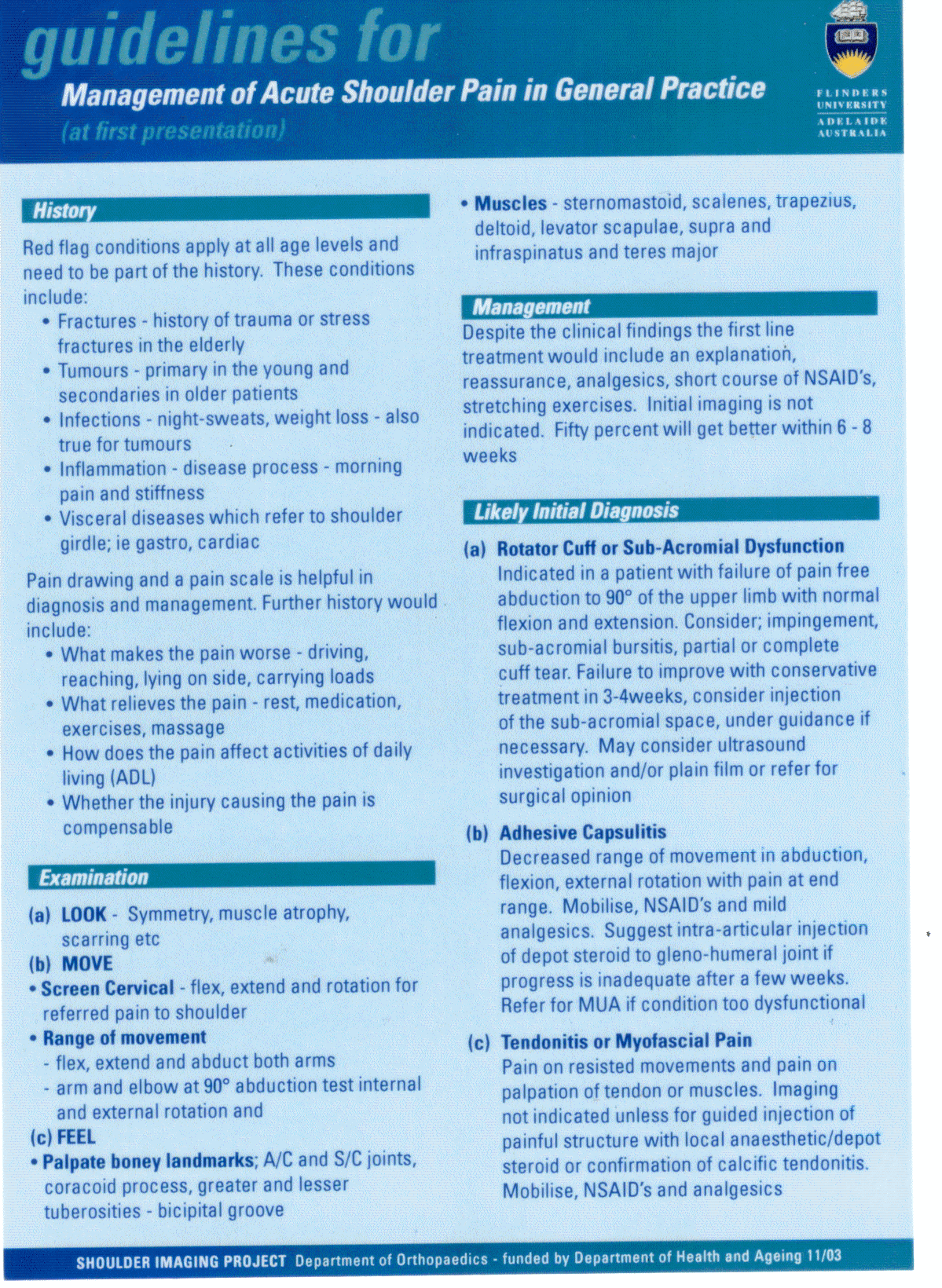

Supplement: Additional File 1 — A guideline for shoulder imaging developed in consensus by 6 orthopaedic surgeons. Guidelines developed for the study for use by GPs to manage shoulder pain. [file 1471-2296-8-12-S1.doc]
