# Supplementary material for: A before and after study of the impact of academic detailing on the use of diagnostic imaging for shoulder complaints in general practice
Source: BMC Fam Pract. 2007 Mar 27;8:12. doi: 10.1186/1471-2296-8-12 (PMC1851961; doi:10.1186/1471-2296-8-12)
Supplement: Additional File 2 — Assessment of knowledge about musculoskeletal problems of the shoulder. The questionnaire used in the study to assess GP knowledge about management of shoulder pain. [file 1471-2296-8-12-S2.doc]

**Assessment of knowledge about musculoskeletal problems of the shoulder.**

Each of the following questions has one best answer. Please circle your choice of best response.

| 1 | When considering a patient with acute non-trauma shoulder pain you would  a) do plain films at first visit  b) take a history and prescribe NSAID’s  c) arrange a referral to an Orthopaedic Surgeon  *****d) make a diagnosis on the basis of history and examination and review in 2 weeks | |
| --- | --- | --- |
| 2 | A ‘red flag’ situation would include  *****a) night sweats in a patient 55 or older  b) failure to respond to your first line of treatment  *****c) pain in the upper limb in an osteoporotic patient following a minor fall  d) failure to progress after 6 weeks in a patient on anti-depressives | |
| 3 | In abduction of the shoulder  *****a) supraspinatus is the initiator followed by trapezius in going from 0-60^0^  b) the same muscles are used when the arm is internally or externally rotated  c) failure to get beyond 90^0^ is impingement until proven otherwise  d) the scapula only begins to move after 90^0^ | |
| 4 | When prescribing NSAID’s for acute MSK pain  a) some NSAID’s are significantly more efficacious than others  b) COX_2_ types have proven to be more efficacious than COX_1_ types  c) nausea and diarrhoea are uncommon side effects  *****d) once a day medication has proven to have a high compliance rate | |
| 5 | | Tests used to diagnose the source of acute shoulder pain  a) have good sensitivity and poor specificity  b) have good specificity and poor sensitivity  *****c) either good sensitivity or specificity but never both  d) the older they are the more reliable they are |
| 6 | | Referred pain to the shoulder girdle from cervical dysfunction usually involves  a) C_1_ nerve root  b) C_3_ nerve root  *****c) C_5_ nerve root  d) C_7_ nerve root |
| 7 | | Which one of the following statements is true  a) pain arising from the long head of biceps can be identified by resisted elbow flexion  b) pain arising from subscapularis enthesis is identified by resisted external rotation  *****c) pain arising from the acromio-clavicular joint occurs after 90^0^ of abduction myofascial  d) pain in the trapezius is readily distinguished from that occurring in supraspinatus |
| 8 | | Which one of the following statements is incorrect  *****a) adhesive capsulitis occurs more commonly in the young (<30)  b) discomfort in the shoulder girdle produces referred pain to the elbow  c) winging of the scapula is due to pathology in the spinal accessory nerve  d) motor supply to trapezius comes from the spinal accessory nerve |
| 9 | | Concerning rotator cuff problems, which of the following is correct  *****a) conservative treatment has a 60% success rate within 3 months  b) shoulder instability does not depend on an intact rotator cuff  c) patients with complete rotator cuff rupture are unable to abduct the arm  d) rotator cuff tears occur at the humeral enthesis |
| 10 | | Impingement relating to movements of the shoulder girdle  a) only refers to the subacromial bursa  b) mainly results from trauma to the gleno-humeral joint  *****c) is best verified using dynamic ultrasound  d) is readily differentiated from other causes of decreased range of movement |

***** Indicates correct answer.

Question 2: either a) of c) is correct
